# Nelfinavir and lenalidomide/dexamethasone in patients with lenalidomide-refractory multiple myeloma. A phase I/II Trial (SAKK 39/10)

**DOI:** 10.1038/s41408-019-0228-2

**Published:** 2019-08-27

**Authors:** F. Hitz, M. Kraus, T. Pabst, D. Hess, L. Besse, T. Silzle, U. Novak, K. Seipel, S. Rondeau, S. Stüdeli, S. Berardi Vilei, P. Samaras, U. Mey, C. Driessen, for the Swiss Group for Clinical Cancer Research SAKK

**Affiliations:** 1 Department of Oncology and Hematology, Kantonsspital St.Gallen, St.Gallen, Switzerland; 2 Department of Medical Oncology, Inselspital, Universitätsspital Bern, Bern, Switzerland; 3 SAKK Coordinating Center, Bern, Switzerland; 4 Hirslanden, Zürich, Switzerland; 5 Department of Oncology, Kantonsspital Graubünden, Chur, Switzerland

**Keywords:** Cancer therapeutic resistance, Cell biology

## Abstract

The antiretroviral agent nelfinavir has antimyeloma activity and can overcome resistance to bortezomib. Our phase I/II trial investigated whether adding nelfinavir to lenalidomide–dexamethasone can overcome lenalidomide resistance in lenalidomide-refractory multiple myeloma (MM). Twenty-nine patients were included (high-risk cytogenetic aberrations 31%; ≥2 prior therapy lines 93%; lenalidomide–bortezomib double-refractory 34%). Twenty-four patients (83%) had prior bortezomib and 10 (34%) were lenalidomide–bortezomib double-refractory. They received four cycles of nelfinavir 2500 mg/day with standard-dose lenalidomide (25 mg days 1–21) and dexamethasone (40/20 mg days 1, 8, 15, 22). Minor response or better was achieved in 16 patients (55%; 95% CI 36–74%), including 40% of those who were lenalidomide–bortezomib double-refractory, and partial response or better in nine patients (31%; 95% CI 15–51%). Median progression-free survival was 3.4 (95% CI 2.0–4.9) months and median overall survival 21.6 (13.0–50.1) months. Lenalidomide-related pneumonitis, pneumonia, and neutropenic fever occurred, but there were no unexpected adverse events. Peripheral blood mononuclear cells showed a 45% (95% CI 40–51%) reduction in total proteasome activity from baseline and significant induction of unfolded protein response and autophagy. Thus, nelfinavir–lenalidomide–dexamethasone is an active oral combination in lenalidomide-refractory MM.

## Introduction

Despite development of new drug combinations, including immunomodulatory drugs (pomalidomide p.o.), proteasome inhibitors (carfilzomib i.v., ixazomib p.o.), and targeted antibodies (daratumumab i.v.), for treatment of relapsed multiple myeloma (MM), the duration of response in double-refractory patients is only 3–5 months^1–3^. Thus, development of alternative myeloma drugs for the refractory setting in particular is an urgent priority. Repurposing of well-known drugs approved for use in other therapeutic settings can potentially provide a shortcut to the development of new myeloma treatments^4^. The best example of this strategy is the success of thalidomide, which now shapes an entire class of myeloma agents, after originally being developed as a hypnotic agent.

Nelfinavir is an oral inhibitor of HIV protease, an enzyme lacking mammalian homologs, and was a standard treatment for HIV infection for several years. It has single-agent activity against myeloma in vivo, inducing cell cycle arrest and apoptosis in tumor cells via multiple pathways, including inhibition of proteasomal degradation and the PI3K/Akt pathway^5–7^ and importantly, by inducing endoplasmic reticulum (ER) stress. In a phase I trial (SAKK 65/08)^8^ we showed that nelfinavir inhibited proteasome activity and significantly upregulated the expression of proteins related to the unfolded protein response (UPR) in peripheral blood mononuclear cells (PMBC). This key modulator of protein production, folding and destruction^9,10^, also determines proteasome inhibitor sensitivity. Combination of nelfinavir with bortezomib further induced the UPR and overcame proteasome inhibitor resistance^8^. A subsequent phase II trial (SAKK 39/13) demonstrated promising clinical activity of a nelfinavir–bortezomib–dexamethasone combination, with an unprecedented overall response rate of 65% in heavily pretreated, proteasome inhibitor–refractory MM patients^11^. Preclinical data indicate that nelfinavir was able to overcome resistance to the proteasome inhibitor carfilzomib via modulation of multidrug resistance (MDR) efflux pumps such as ABCB1 (ref. ^12^). As lenalidomide is a substrate of the same pump family^12^, and via binding to the E3 ligase cereblon, works on the ubiquitin–proteasome system, we postulated that nelfinavir might likewise be able to overcome resistance to immunomodulatory drugs.

Thus, we conducted a phase I/II study to determine whether adding nelfinavir to lenalidomide–dexamethasone (Len-Dex) can overcome lenalidomide resistance in patients with lenalidomide-refractory MM. Results from this study have been reported previously in abstract form^13^.

## Patients and methods

Patients with lenalidomide-refractory MM, defined according to IMWG criteria (MM that had progressed during or within 60 days after termination of lenalidomide-containing therapy of ≥2 months’ duration)^14^, were eligible for the study. They were required to have measurable disease (serum M-protein ≥10 g/L for IgG or ≥5 g/L for IgA, IgM, or IgD, or urine M-protein ≥200 mg/24 h) or in the absence of measurable disease, serum immunoglobulin free light chain >100 mg/L^15^. Also required were adequate hematologic values (neutrophils ≥1 × 10^9^/L and platelets ≥75 × 10^9^/L) and hepatic function (bilirubin ≤ 1.5 × ULN, AST, and AP ≤ 2.5 × ULN), as well as calculated creatinine clearance >50 mL/min and cardiac ejection fraction ≥40%. Excluded were patients receiving potent CYP3A4 modulators during the study and those with previous grade 4 adverse events related to lenalidomide: previous malignancy, HIV positivity, psychiatric disorders, uncontrolled cardiac disease, or a serious underlying medical condition.

All patients provided written informed consent. The trial was conducted according to applicable national and international laws and regulations and approved by regulatory authorities (registered at ClinicalTrials.gov: NCT01555281).

Len-Dex was administered according to a standard schedule (Len 25 mg p.o. days 1–21; Dex 20/40 mg p.o. days 1, 8, 15, 22). In phase I, a standard 3 + 3 dose escalation design was used to identify the recommended phase II dose (RP2D) of nelfinavir for use in combination with Len-Dex. The nelfinavir dose-escalation scheme was 1250 mg orally b.i.d. (2500 mg daily) for dose level (DL) 1 and 1875 mg b.i.d. (3750 mg daily) for DL 2. Nelfinavir was taken from days 1 to 21 of every cycle. The decision to escalate to the next DL was based on the number of dose-limiting toxicities (DLTs) observed in the first cycle of trial treatment. Persistent and late toxicities were also considered.

In phase II nelfinavir was administered at the RP2D in combination with Len-Dex (as above) for four cycles or until progression, unacceptable toxicity, or patient withdrawal. Response was assessed at the start of each treatment cycle and confirmed by a central committee. In addition to standard IWIG response criteria^14^ [complete response (CR): very good partial response (VGPR), and partial response (PR)], we included minor response (MR), as defined by Kyle and Rajkumar:^15^ a ≥25% but <49% reduction of serum M protein *and* reduction in 24 h urine M protein by 50–89%, which still exceeds 200 mg/24 h and a 25–49% reduction in the size of soft tissue plasmacytomas (if present at baseline) and no increase in the size or number of lytic bone lesions. This response category is used in patients with relapsed/refractory MM to obtain a signal of activity in phase I/II trials of novel agents. The primary endpoint in phase II was best response (CR, VGPR, PR, or MR) within 16 weeks (4 cycles).

Toxicities were graded according to the National Cancer Institute Common Terminology Criteria for Adverse Events (CTCAE) v.4.0.

### Pharmacodynamics

Whole-blood samples were obtained from seven patients at baseline, day 8 and day 15 of the phase I study. PBMC were isolated and cell lysates used to test for pharmacodynamic markers: proteasome activity was measured in-gel after SDS-PAGE as described previously^16^ using a proteasome-specific, active site-directed fluorescent chemical probe^17^ (kindly supplied by Herman Overkleeft, Leiden University), which visualizes both immuno- and constitutive proteasome subunit activities. Expression of UPR-associated proteins was evaluated using western blot techniques after SDS-PAGE: blots for phospho-(S724) IRE1α (Abcam, Cambridge, UK) to evaluate activation of the main ER stress axis IRE/XBP and blots for CCAAT-enhancer-binding protein homologous protein (CHOP) (Cell Signaling Technology, Danvers, USA) to show UPR leading to ER stress-induced apoptosis. Blots against LC3A (Cell Signaling Technology, Danvers, USA) were used to evaluate autophagy. GAPDH (Proteintech, Manchester, UK) served as a loading control. Quantitative assessments were calculated relative to loading control using densitometry.

For the determination of proteasome activity, the fluorescent-labeled activity-based proteasome probe (MV151) was incubated for 1 h at 37 °C with PBMC lysates. Subsequently, the samples were denatured for 2 min at 95 °C and separated by polyacrylamide gel electrophoresis. Proteasome activity was visualized via quantitative assessment of the respective specific fluorescence signals using Fusion Solo S Western Blot and Chemi Imaging System (Vilber).

### Statistical considerations

In phase II, the Simon’s two-stage design^18^ was used to test the null hypothesis of a response rate (MR or better) ≤10% versus the alternative hypothesis of a response rate ≥30%. With a one-sided significance level of 5% and a power of 80%, a total of 29 patients (including the 6 patients from phase I treated at the RP2D) were required with 10 patients in the first stage and 19 more patients in the second stage (calculated using PASS 2011, by NCSS, Kaysville, USA). At the second-stage analysis, if at least six patients had achieved MR or better the null hypothesis was to be rejected and the trial regimen considered active and promising for further investigation.

The OR rate and corresponding Clopper–Pearson 95% confidence interval (CI) were calculated. For time-to-event endpoints median values, along with 95% CI, were estimated using the Kaplan–Meier (KM) method. The Wilcoxon signed-rank test was used to determine differences from baseline in pharmacodynamic parameters. Statistical analyses were performed using SAS® 9.4 (SAS Institute Inc., Cary, NC, USA).

## Results

### Patients

Between May 2012 and December 2016, 29 patients were enrolled at seven Swiss centers. Ten patients were included in phase I and analyzed for DLTs: two DLTs were observed: diarrhea grade 3 and thrombocytopenia grade 4 at a dose level of 1850 mg b.i.d. Nelfinavir 1250 mg b.i.d. (2500 mg daily) was therefore identified as the R2PD. Twenty-nine patients were included in phase II, including six patients from phase I.

Patient and disease characteristics for the 29 patients are presented in Table 1. Most (93%) had undergone two or more prior lines of therapy, and 24 (83%) had prior bortezomib exposure, including 18 (62%) with prior autologous stem cell transplantation and 10 (34%) who were double-refractory to lenalidomide and bortezomib, based on International Myeloma Working Group (IMWG) criteria^14^.

**Table 1:** Patient characteristics

	Total (*n* = 29)
**Age (years)**	64 (40–82)
**Sex**	
Female	11 (38%)
Male	18 (62%)
**Performance status**	
0	16 (55%)
1	12 (41%)
2	1 (3%)
**Clinically significant concomitant disease**	19 (66%)
**ISS stage**	
I	10 (34%)
II	11 (38%)
III	8 (28%)
**High-risk cytogenetic aberrations t(4;16), t(14;16), del17p**	9 (31%)
**Previous regimens**	
Number of prior lines	2 (1–7)
≥2 prior lines	27 (93%)
Autologous stem cell transplantation	18 (62%)
**Lenalidomide exposure**	
Refractory	29 (100%)
immediate previous line of lenalidomide therapy	20 (69%)
any previous timepoint of lenalidomide therapy	9 (31%)
Progressive	
While on lenalidomide therapy	24 (83%)
Within 60 days of cessation of lenalidomide therapy	5 (17%)
Median dose of lenalidomide in the last cycle prior to enrollment (mg)	25 (10–25)
**Lenalidomide** **+** **bortezomib exposure**	
Exposed	24 (83%)
Refractory	10 (34%)

Data are *n* (%) or median (range)

Fifteen patients completed four cycles of study treatment. Fourteen of 29 patients discontinued trial treatment due to: progressive disease (PD; *n* = 8), unacceptable toxicity (4), or patient refusal (2).

### Response and survival

Sixteen patients (55%; 95% CI 36–74%) achieved a response (MR or better), with VGPR in three patients (10%), PR in six patients (21%), and MR in seven patients (24%). Of the remaining patients, four (14%) had stable disease (SD), seven (24%) had PD, and two patients were not evaluable for response analysis. Subgroup analysis revealed seven responders among the nine patients with high-risk cytogenetic features (78%: 1 VGPR/2 PR/4 MR) and four responders among the 10 patients with double-refractory disease (40%; 1 VGPR/1 PR/2 MR).

Partial response or better was achieved in 9 (31%; 95% CI 15–51%) of patients.

Four responding patients received additional cycles (*n* = 2: 3 patients; *n* = 7: 1 patient) of trial treatment on a compassionate-use basis after completing the trial.

After a median follow-up of 24.9 months (interquartile range 21.8–28.6 months), median progression-free survival was 3.4 (95% CI 2.0–4.9) months, overall duration of response 4 (1.8–5.7) months, and median overall survival 21.6 (13.0–50.1) months (Table 2).

**Table 2:** Response and survival outcomes (*n* = 29 patients)

**Best response** **≥** **MR**	16 (55%, 95% CI 36–74)
**Best response categories**	
Very good partial response (VGPR)	3 (10%)
Partial response (PR)	6 (21%)
Minor response (MR)	7 (24%)
Stable disease (SD)	4 (14%)
Progressive disease (PD)	7 (24%)
Not evaluable	2 (7%)
**Best response** **≥** **MR by subgroup**	
High-risk cytogenetics (n = 9)	7 (78%)
VGPR	1
PR	2
MR	4
Lenalidomide + bortezomib; double-refractory (n = 10)	4 (40%)
VGPR	1
PR	1
MR	2
**Duration of response (months) (*n*** = 16)	4.0 (1.8–5.7)
**Overall survival (OS), median (95% CI) (months)**	21.6 (13.0–50.1)
6-month OS rate	82% (62–92)
12-month OS rate	79% (58–90)
18-month OS rate	59% (38–75)
24-month OS rate	45% 25–63)
**Time to progression, median (95% CI) (months)**	3.6 (2.0–5.1)
**Progression-free survival (PFS), median (95% CI) (months)**	3.4 (2.0–4.9)
4-month PFS rate	43% (25–60)
6-month PFS rate	20% (7–36)
12-month PFS rate	8% (1–22)

Data are shown as *n* (%), median (95% CI) or % (95% CI) unless otherwise indicated. Median follow-up 24.9 months

### Tolerability

The most frequent adverse events were grade 1 gastrointestinal symptoms (nine patients) and metabolic disorders (nine patients). Hematologic adverse events grade ≥3 were anemia (seven patients), thrombocytopenia (six patients), and neutropenia (seven patients, including two patients with febrile neutropenia). Patients with neutropenic fever were allowed to receive treatment with granulocyte-colony-stimulating factor.

Non-hematologic adverse events grade ≥3 were dyspnea (three patients; unrelated to study treatment) and bone pain grade 3 (two patients; related to traumatic iliac fracture in the absence of progressive disease in one patient). Other non-hematologic events, all judged to be possibly/probably related to lenalidomide, were lung infection (three patients; *n* = 1 grade 3/1 grade 5; proven respiratory syncytial virus infection), pneumonitis (1 patient; grade 2), and fatigue, a well-known adverse effect of both lenalidomide and nelfinavir (13 patients; *n* = 3 grade 3).

### Pharmacodynamics

**Fig. 1:**
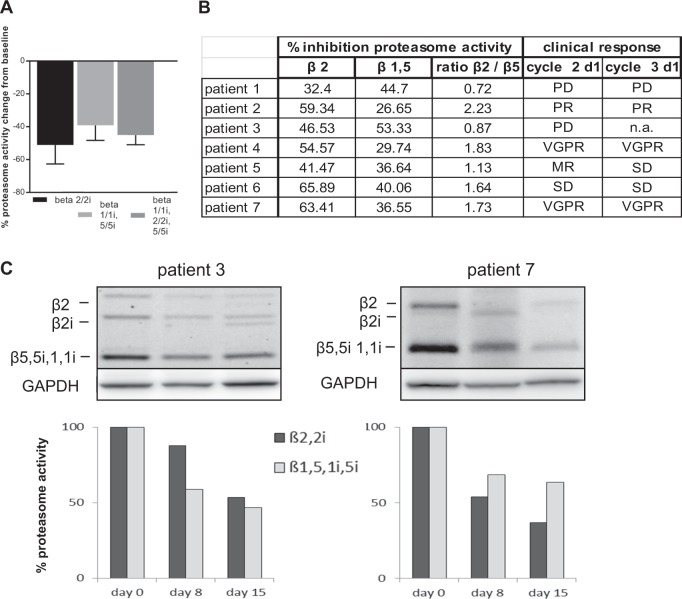
Proteasome activity in PBMC. **a** Maximum change from baseline in proteasome activity on days 8 or 15 (β2/2i, β1/1i, 5/5i proteasome subunits and global proteasome activity), mean of all patients. **b** Comparison of β2/2i/β1/1i, 5/5i ratio versus clinical response. **c** Active site labeling days 0 (baseline), 8, and 15 (*n* = 2 patients) and its densitometric evaluation of the bands

The mechanism of action of nelfinavir in myeloma therapy has not been identified. However, high concentrations provide proteasome inhibition in vitro^16^. Lenalidomide has synergistic activity with proteasome inhibitors in vivo^19^. Activity-based chemical tools were developed that allow for a direct, specific, activity-dependent visualization of active proteasome subunits^17^. We used this tool to assess proteasome activity and related pharmacodynamics parameters in a set of PBMC samples from seven treated patients taken at baseline, day 8, and day 15 of cycle 1. We observed a significant (*p* = 0.0156) reduction in proteasome activity in vivo, with a 51% inhibition in β2/2i (tryptic) activity, 39% in β1/1i, 5/5i (caspase-like/chymotryptic, bortezomib-type inhibition pattern), and 45% (95% CI 40–51%) in pan-proteasome activity of all proteasome subunits (marizomib-type inhibition pattern) (Fig. 1a). Most recent evidence suggests that co-inhibition of β2/2i proteasome activity, in addition to inhibition of β5/β1, is key to the activity of proteasome inhibitors against refractory MM^20^. We therefore relate the β2/2i versus β1/1i,5/5i ratios to the clinical outcome of the seven patients: the two patients who did not respond to treatment showed relatively poor β2/2i co-inhibition, with a β2/β1–β5 ratio <1 (Fig. 1b). Figure 1c shows activity-based proteasome labeling in PBMC from two representative patients and corresponding densitometric analysis.

**Fig. 2:**
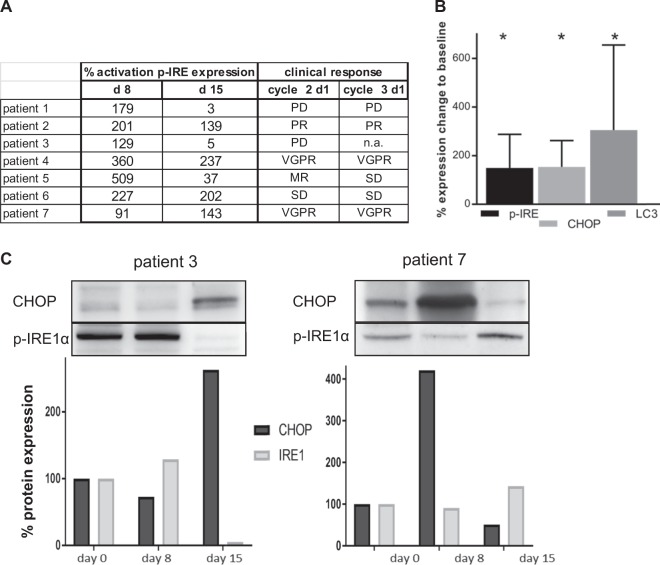
UPR and autophagy in PBMC. **a** Comparison of phospho-IRE1α expression versus clinical response. **b** Maximum change from baseline in UPR, western blots densitometric evaluation (phospho-IRE1α, CHOP), and autophagy (LC3A) [mean of all patients]. **c** Western blots for ER stress proteins (phospho-IRE1α, CHOP) and densitometric evaluation on days 0, 8, and 15 from two representative patients

Proteasome inhibition results in myeloma cell death through the induction of excess proteotoxic stress that manifests in an upregulation of UPR-related proteins and the induction of autophagy. In Fig. 2a the expression of the ER stress protein phospho-IRE1α on day 8 and 15 is shown relative to the clinical outcome of the 7 patients. Interestingly, patients who had PD as best clinical response did not exhibit increased UPR activity on day 15, while those who maintained a substantial upregulation of phospho-IRE1α (>100% of baseline value) responded to treatment or showed SD. In Fig. 2b we show the densitometric evaluation of western blots: mean expression of all patients (maximum change versus before treatment) for LC3 (mean of 305%, *p* = 0.031), indicating increased autophagy, for CHOP (mean of 254%, *p* = 0.016), and for activated phospho-IRE1α (to a mean of 250%, *p* = 0.016), both indicating elevated ER stress. Two representative PBMC samples are shown in Fig. 2c.

## Discussion

Our phase II study shows that a combination of standard Len-Dex with nelfinavir is active in patients with lenalidomide-refractory MM, including those with double-refractory disease. Thus, the combination of nelfinavir–lenalidomide–dexamethasone overcomes lenalidomide drug resistance in a relevant proportion of patients. We reported earlier that the combination of nelfinavir with bortezomib–dexamethasone achieved a noteworthy 65% ORR in patients with double-refractory MM^11^. Given the established safety, worldwide availability, oral route of administration, and competitive costs of nelfinavir, together with its FDA-recognized status as an orphan drug for MM, we continue to explore the clinical potential of re-purposing nelfinavir for the treatment of patients with heavily pretreated, refractory disease.

Nelfinavir–lenalidomide–dexamethasone achieved a response rate of 55% (MR or better, 95% CI 36–74%) in our patients with lenalidomide-refractory MM, with an ORR of 31% (95% CI 15–51%) (i.e PR or better). High rates of response were seen in patients with high-risk cytogenetic aberrations (78% MR or better) and those who were double-refractory to previous lenalidomide and bortezomib treatment (40% MR or better). The duration of response was 4 months in this heavily pretreated patient population. Although the patient numbers in this study are low and preclude definite conclusions, the response rates are in a similar order of magnitude to the benchmark for next-line active therapy in double-refractory MM^21^. Moreover, because of the short duration of this study it is likely that the clinical benefit of the combination was underestimated—patients could receive a maximum of only four cycles, since there was limited external funding support for this non-commercial trial. Treatment was well tolerated, with hematologic and non-hematologic toxicities consistent with the expected side effects of the individual components. Although patients had significant comorbidities, adverse events were manageable with optimized supportive care. Nevertheless, a small number of severe adverse events, such as a grade 2 pneumonitis, were encountered. Lung toxicity is a well-known complication of lenalidomide, but a potential contribution of nelfinavir cannot be excluded.

Active therapeutic options in the lenalidomide-refractory, and in particular in the proteasome inhibitor-lenalidomide double-refractory MM setting, remain limited. Such patients were excluded from drug approval trials evaluating triplets of ixazomib, daratumumab, or carfilzomib in combination with lenalidomide/dexamethasone. Carfilzomib achieved a clinical benefit rate (≥MR) of 37%^22^, daratumumab 37%^23^, and pomalidomide 39%^2^ in heavily pretreated refractory MM patients. Lenalidomide likewise showed synergistic activity with continuous low-dose cyclophosphamide, with ≥MR in 83% of patients, including those with lenalidomide- and bortezomib-refractory disease and high-risk cytogenetic abnormalities^24^.

Further studies evaluating the activity of Len-containing triplets in Len-refractory patients are very scarce. Len-Dex combined with daratumumab in a phase III trial demonstrated a very high ORR of 92.9%, versus 76.4% for the Len-Dex control group (P < 0.001). The study population included patients exposed to lenalidomide but only 3.5% were refractory in previous lines of therapy^25^.

Given the lack of hematotoxicity of nelfinavir, it is tempting to speculate that a quadruplet regimen of lenalidomide, nelfinavir, cyclophosphamide, and dexamethasone might potentially provide an even more active oral treatment option for patients with drug-refractory MM. Pomalidomide-based triplets (Elotuzumab + Pom/Dex, Daratumumab + Pom/Dex) have recently been explored and appear to have a similar or better response profile than the Nelfinavir + Len/Dex regimen investigated here^26,27^. However, the Nelfinavir + Len/Dex regimen is one of the very few all-oral combinations that can provide stabilization of the disease for a majority of patients in the relapsed-refractory setting. Moreover, the components are available and affordable even in countries with low/intermediate incomes, in contrast to triplets containing pomalidomide and antibodies.

The mechanism of activity of nelfinavir against MM is not yet well established. Employing last-generation chemical biology-based tools that directly address proteasome subunit activity in patient-derived cells, we observed a significant inhibition of proteasome activity and subsequent upregulation of markers of proteotoxic stress in PBMC from patients treated with nelfinavir–lenalidomide–dexamethasone in our study. While the number of samples is small, this finding suggests that synergy between functional proteasome inhibition and the activity of lenalidomide might underlie the clinical activity of the study combination. Indeed, the degree of in vivo proteasome inhibition we observed in PBMC during study treatment, at approximately 50% versus baseline, is quantitatively quite relevant, since inhibition achieved with standard bortezomib treatment is in the 60–70% range in clinical trials^28^. Ixazomib, the only oral proteasome inhibitor approved to date, has significantly lower proteasome-inhibiting activity than bortezomib, with an IC_50_ of 1000 nM for β5, respectively in vitro^20^ and, to our knowledge, in vivo activity is yet to be reported. The epoxyketone next-generation oral proteasome inhibitor oprozomib has shown poor tolerability, with marked gastrointestinal side effects^29^.

The nelfnavir related HIV protease inhibitor ritonavir likewise induces proteasome inhibition at high concentrations, by interfering with regulatory subunits of the proteasome^16^. Nelfinavir has pan-proteasome-inhibiting activity at high concentrations (20–40 μM) in vitro^30^, but peak nelfinavir concentrations at the 2 × 1875 mg dose are in the 15 μM range in treated patients^8^. We speculate that adding lenalidomide to nelfinavir treatment may increase intracellular nelfinavir concentrations sufficiently to mediate the pan-proteasome-inhibiting activity seen with high concentrations of nelfinavir. Both nelfinavir and lenalidomide are substrates of MDR-1 type drug efflux pumps^12^; thus, competing interaction of the two drugs for the MDR-1 drug exporter may limit the export of nelfinavir in the presence of lenalidomide. This theory is consistent with the observation that the MTD of nelfinavir in combination with bortezomib (a poor MDR-1 substrate) and dexamethasone was 2 × 2500 mg/day (ref. ^8^), while in our present study the MTD was only 2 × 1250 mg in combination with lenalidomide–dexamethasone in a comparable population of MM patients. Likewise, nelfinavir may have contributed to the clinical activity of the combination therapy by increasing intracellular lenalidomide concentrations within myeloma cells^12^.

Comparison of pharmacodynamic data with clinical outcomes in our study suggests that a relatively high proportion of β2/2i inhibition and prolonged induction of a proteotoxic stress response are associated with clinical response. This is in line with the most recent notion that co-inhibition of β2-type proteasome activity is particularly important for the cytotoxic activity of proteasome inhibitors in the drug-refractory setting, leading to enhanced induction of proteotoxic stress, as seen with high-dose (but not low-dose) carfilzomib^20^.

## Conclusion

A combination of nelfinavir with Len/Dex was found to be active in patients with lenalidomide-refractory myeloma, including those with lenalidomide–bortezomib double-refractory and high-risk disease. New treatments are urgently needed for such patients. The combination treatment resulted in significant proteasome inhibition and induction of proteotoxic stress in vivo. This new regimen offers the advantages of all-oral administration and lower costs than those based on novel proteasome-targeted drugs, given that nelfinavir is available in generic form. These findings warrant further evaluation of nelfinavir in MM.
